# Bacterial Degradation of Antinutrients in Foods: The Genomic Insight

**DOI:** 10.3390/foods13152408

**Published:** 2024-07-29

**Authors:** Alexander Arsov, Lidia Tsigoriyna, Daniela Batovska, Nadya Armenova, Wanmeng Mu, Wenli Zhang, Kaloyan Petrov, Penka Petrova

**Affiliations:** 1 Institute of Microbiology, Bulgarian Academy of Sciences, 1113 Sofia, Bulgaria; al.arsov@microbio.bas.bg; 2 Institute of Chemical Engineering, Bulgarian Academy of Sciences, 1113 Sofia, Bulgaria; lidinka29@gmail.com (L.T.); danielabatovska@gmail.com (D.B.); nadq.armenova@gmail.com (N.A.); kaloian04@yahoo.com (K.P.); 3 State Key Laboratory of Food Science and Resources, Jiangnan University, Wuxi 214122, China; wmmu@jiangnan.edu.cn (W.M.); wenlizhang@jiangnan.edu.cn (W.Z.)

**Keywords:** antinutrients, phytic acid, oxalates, tannins, saponins, lectins, cyanogenic glycosides, protease inhibitors, fermentation

## Abstract

Antinutrients, also known as anti-nutritional factors (ANFs), are compounds found in many plant-based foods that can limit the bioavailability of nutrients or can act as precursors to toxic substances. ANFs have controversial effects on human health, depending mainly on their concentration. While the positive effects of these compounds are well documented, the dangers they pose and the approaches to avoid them have not been discussed to the same extent. There is no dispute that many ANFs negatively alter the absorption of vitamins, minerals, and proteins in addition to inhibiting some enzyme activities, thus negatively affecting the bioavailability of nutrients in the human body. This review discusses the chemical properties, plant bioavailability, and deleterious effects of anti-minerals (phytates and oxalates), glycosides (cyanogenic glycosides and saponins), polyphenols (tannins), and proteinaceous ANFs (enzyme inhibitors and lectins). The focus of this study is on the possibility of controlling the amount of ANF in food through fermentation. An overview of the most common biochemical pathways for their microbial reduction is provided, showing the genetic basis of these phenomena, including the active enzymes, the optimal conditions of action, and some data on the regulation of their synthesis.

## 1. Introduction

Antinutrients or anti-nutritional factors (ANFs) are plant food constituents that can adversely affect the intake or assimilation of essential nutrients or could form toxic compounds during their breakdown [1,2,3]. These ingredients have such a controversial impact on human health that, although some authors suggest potential beneficial effects, others recommend particularly careful consumption of foods containing them, and only after processing [3,4,5,6,7,8]. There are several studies reporting antioxidant, anti-inflammatory, anti-obesity, immunomodulatory, and cardioprotective properties of ANFs [9,10]. Protease inhibitors and saponins, two major classes of ANFs, are strongly associated with anticarcinogenic effects [1,11,12]; however, some synthetic saponins with high activity against tumor cells have shown comparable toxicity to normal cells [13].

Without neglecting the need to gather new scientific data, our current knowledge makes it clear that the positive effects of antinutrients are probable but have yet to be elucidated [11], while their negative ones are beyond a reasonable doubt [14,15,16,17]. In the short term, ANFs can decrease the bioavailability of essential nutrients such as metal ions, amino acids, lipids, and vitamins [2,3]. In the long term, their daily presence in the diet can lead to systemic and severe health issues, including impaired intestinal integrity, kidney stones, low plasma levels of iron and calcium (hyposideremia and hypocalcemia), as well as immunological disorders such as the non-celiac wheat sensitivity (NCWS) that shares the symptoms of celiac disease and irritable bowel syndrome [4,18]. Therefore, the Paracelsus dictum “the dose makes the poison” applies to antinutrients as well as to most plant substances with medicinal properties.

In an effort to reduce ANF in many staple foods, agriculture has sought to select plants with lower antinutrient content and to develop various approaches for microbial fermentation of plant food for this purpose [19]. The fine balance of ANF intake in safe amounts is already controlled by the microbial communities processing sourdough, cassava, sorghum, lentils, legumes, and many pseudocereals. Over the last decade, there has been a plethora of studies that highlight the fermentation by dietary (or probiotic) microorganisms as a major approach to reducing the amounts of phytates, cyanogenic glucosides, and amylase and trypsin inhibitors in fermented foods [10,20,21].

This review aims to summarize the scientific research in the area to date and to describe our current understanding of the genetic and biochemical mechanisms behind microbial reduction in antinutrients in fermented foods. The chemical structure of ANFs, their abundance in certain foods, and their adverse effects on human health are also discussed below.

## 2. Classification, Abundance and Adverse Health Effects of Antinutrients

**Figure 1:**
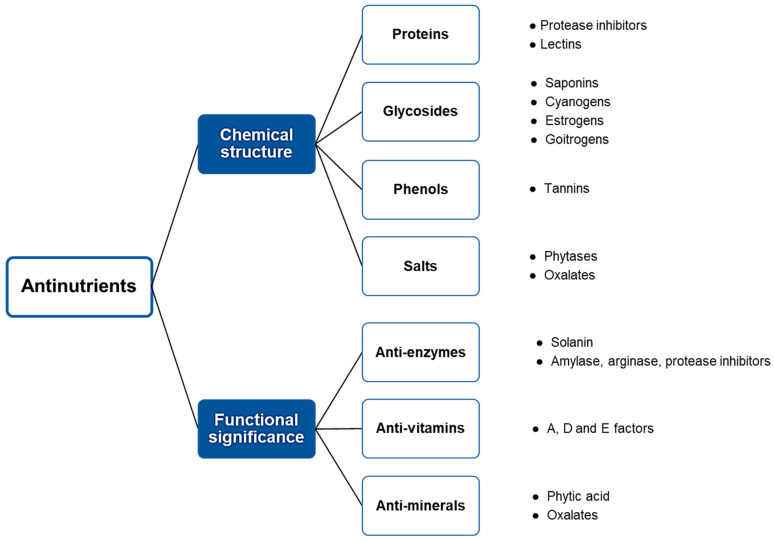
Classification of antinutrients in foods by their chemical structure and functional significance.

Antinutrients can be classified into two groups based on their sensitivity to heat: heat-stable antinutrients and heat-labile antinutrients [22,23]. The heat-stable antinutrients include phytic acid, tannins, alkaloids, saponins, and non-protein amino acids, while the heat-labile antinutrients include lectins, cyanogenic glycosides, and protease inhibitors [2,3,4]. Another classification (shown in Figure 1) combines chemical structure (proteins, glycosides, phenols) with functional significance (anti-minerals, anti-vitamins, anti-enzymes).

A brief overview of the antinutrient factors (ANFs) discussed below, the foods that contain them in high amounts, and the most common adverse effects on human health are presented in Table 1.

### 2.1. Antinutrients with Protein Structure

ANFs, which are proteins, include (i) the protease inhibitors, especially of digestive enzymes such as trypsin; and (ii) lectins, carbohydrate-binding proteins widely distributed in the legume family and amounting to 2–10% of the total protein in legume seeds [24].

Plant protease inhibitors (PPIs) are widely distributed in the plant kingdom and ubiquitous in the *Solanaceae*, *Fabaceae*, *Euphorbiaceae*, *Poaceae*, and *Cucurbitaceae* families [25]. Typical plants rich in PPIs are soybean (*G. max*), rice (*Oryza sativa*), sorghum (*Sorghum bicolor*), wheat (*Triticum aestivum*), barley (*Hordeum vulgare*), potato meals (*Solanum tuberosum*), red gram (*Cajanus cajan*), chickpea (*C. arietinum*), lima bean (*P. lunatus*), cowpeas (*Vigna unguiculata*), peas (*Pisum sativum*), and lentils (*Lens culinaris*) [26,27].

Bowman–Birk and Kunitz-type inhibitors are the best-known PPI families [28]. The first type of PPIs has a molecular weight of 4 to 8 kDa, one or more inhibitory domains, and is highly effective against trypsin, whereas the second type has a molecular weight of 18 to 24 kDa, single or double inhibitory domains, and inhibits both trypsin and chymotrypsin [29,30]. Both PPI types function as competitive inhibitors. Due to their proteolysis regulatory function, many have been found promising agents for obesity, cardiovascular diseases, autoimmune disease treatment, alleviation of inflammatory processes, some types of cancer, and neurodegenerative diseases [31,32]. Despite this fact, PPIs are classified as antinutrients. They bind to the active site of human and animal protein-digesting enzymes, creating a complex with a low dissociation constant that inhibits protease activity [1]. This is followed by the activation of pancreatic trypsin and chymotrypsin secretion, which increases the demand for sulfur-containing amino acids like methionine and cysteine, resulting in nitrogen and sulfur loss [27]. Intake of trypsin/chymotrypsin inhibitors temporarily halts the digestion of food, resulting in poor protein absorption, delayed growth, muscle mass loss, increased bile output, gastric anguish, pancreatic hypertrophy, and hyperplasia [26,31,33].

Lectins are proteins or glycoproteins characterized by their ability to bind carbohydrates, and they can be grouped according to their origin—animal, algal, bacterial, fungal, and plant lectins [34,35]. More than 500 lectin variants have been isolated; they are also known as “hemagglutinins” because of their ability to bind and agglutinate erythrocytes [2,24]. Animal lectins primarily target carbohydrate complex structures; algal lectins bind specifically to glycoproteins; bacterial lectins have an affinity for glycans; fungal lectins are attracted to N-acetyl galactosamine; and plant lectins typically recognize monosaccharides and oligosaccharides [36,37,38,39,40]. Lectins can be classified based on their affinity for specific carbohydrates into five groups: (i) glucose/mannose; (ii) galactose and N-acetyl-D-galactosamine; (iii) N-acetylglucosamine; (iv) L-fucose; and (v) sialic acid. This classification reflects the specific carbohydrate structures that lectins bind to, which is essential for their diverse biological functions and potential therapeutic applications [41]. In plants, they can be found in wheat, nuts, cereals, beans, quinoa, peas, and mainly in leguminous seeds [42]. Since lectins are relatively resistant to the activity of digestive enzymes in the gastrointestinal tract, they can remain intact as they pass through the digestive system. Hence, their presence can be related to diseases such as colitis, Crohn’s disease, Coeliac-Sprue, and IBS [41,43]. On a molecular level, lectins can induce caspase-1 activation and IL-1β secretion via the NLRP3 inflammasome, thus promoting inflammatory disorders. On a subcellular level, lectins are internalized into the endoplasmic reticulum and then trigger Ca^2+^ release coupled with mitochondrial damage [44]. When the body is exposed to large amounts of lectins, a condition known as leaky gut syndrome occurs, including an increase in intestinal permeability, where the lining of the intestine becomes more porous, allowing substances such as toxins, undigested food particles, and bacteria to leak into the bloodstream. This leakage triggers an immune response and can lead to inflammation throughout the body [45]. Lectins can also mimic the action of insulin on target cells and even trigger the release of insulin by the pancreas. This can potentially disrupt normal insulin signaling and blood sugar regulation [46]. According to Singh et al. [41], lectins are responsible for acne, inflammation, migraines, and joint pains, and have also been implicated in auto-immune diseases [47].

Some lectins, such as ricin and abrin in particular, are very toxic. Food poisoning due to poorly cooked beans rich in lectins is extensively documented in China and the UK [48,49]; their toxicity to numerous insect pests has made them potential insecticides [50]. In recent years, despite concerns about the adverse effects associated with consuming unprocessed foods containing lectins, various studies have highlighted the therapeutic potential of lectins in diagnosing and treating several diseases. Notably, lectins have shown promise in cancer treatment due to their potential antiangiogenic, antimetastatic, and antiproliferative activities [51]. Lectins have also been shown to possess antibacterial, antimicrobial, antiviral, and antifungal properties [52]. The dual nature of lectins, having both potentially harmful and therapeutic benefits, highlights the importance of careful handling and the thorough research needed on the topic to maximize their medical utility while ensuring safety.

### 2.2. Glycosides

Glycosides include (i) cyanogenic glycosides (e.g., amygdalin, linamarin, dhurrin, prunasin, taxiphyllin), which are α-hydroxynitriles glycosylated with glucose or gentiobiose (disaccharide of two glucose moieties joined with a β(1→6) bond) [53,54]; (ii) goitrogens, present in turnip, cabbage, and sweet potato, a diverse group of about 120 glucosinolates (derivatives of glucose and amino acids); and (iii) saponins, foam-producing glycosides with triterpene or steroid backbone and a wide range of sugars attached to them, widely distributed in the plant kingdom, notably in legumes (triterpenoid version) and oats, pepper, asparagus, and ginseng, among others (steroid version) [55]. In general, glycosides are made up of a carbohydrate moiety (glycone) and a non-carbohydrate moiety (aglycone) linked by an ether bond [56]. Both moieties differ greatly in structure and function. Cyanogenic glycosides have an aglycone of the α-hydroxynitrile type, whereas saponins have an aglycone with a steroid or triterpenoid skeleton [57,58,59,60].

Cassava, a staple food in Africa but also popular in South America and South-East Asia, is the most cyanogenic of all edible plants. All parts (leaves, stem, root) contain appreciable amounts of cyanogenic glycosides, which are also found in cocoyam (leaves, roots), sorghum (fruits, leaves, shoot tips), bamboo (stem, sprouts), apples (seeds and fruits), and apricots (kernels) [61].

Cyanogenic glycosides are amino acid-derived secondary metabolites present in over 2500 plant species, primarily in the *Fabaceae*, *Rosaceae*, *Linaceae*, *Poaceae*, and *Compositae* families [62,63,64]. These chemicals are widely occurring in the edible seeds and fruits of plants, including apples (*Malus sylvestris*), apricots (*Prunus armeniaca*), cherries (*Prunus avium*), peaches (*Prunus persica*), plums (*Prunus domestica*), and quinces (*Cydonia oblonga*) [22,62,64]. They are also abundant in almonds (*Amygdalus communis*), cashews (*Anacardium occidentale*), cassava (*Manihot esculenta*), bamboo shoots (*Bambusa vulgaris* and *Phyllostachys edulis*), linseed (*Linum usitatissmium*), cocoyam (*Colocasia esculenta*), lima beans (*Phaseolus lunatus*), chickpeas (*Cicer arietinum*), sorghum (*Sorghum bicolor*), and barley (*Hordeum vulgare*) [22,59,63,65]. Other food products that may include cyanogenic glycosides include flavorings such as almond powder or paste, marzipan, stone fruit (*Prunus* spp.), and stone fruit-based alcoholic drinks [62]. The number of cyanogenic glycosides produced is determined by the plant’s age, variety, and environmental conditions [56]. When hydrolyzed, these ANF generate hydrogen cyanide HCN, a potent respiratory inhibitor. A single dose of cyanide of about 1–3 mg/kg is lethal for most vertebrates. For instance, cassava is a staple food high enough in cyanogens to be a real health hazard if not properly processed [53]. Cyanide poisoning, whether acute or chronic, is rare due to the convergence of circumstances [56]. But there is some evidence that chronic exposure to dietary cyanide from poorly processed cassava may be a factor in growth retardation in children [66]. Chewing and processing cyanogenic plants disrupt their cell structures, which results in the hydrolysis of cyanogenic glycosides by plant β-glucosidase [67]. This process produces sugars and cyanohydrin, which break down spontaneously into hydrogen cyanide (HCN) and the respective aldehyde or ketone [68]. The release of HCN, also known as a “cyanide bomb”, makes an effective chemical defense against herbivores and pathogens [56,69]. However, it may cause dangerous medical conditions in livestock and humans. Cyanide prevents the human body from using oxygen and speeds up anaerobic metabolism, which causes an overabundance of lactic acid, metabolic acidosis, and ultimately cell death [57,65]. The range of 0.5 to 1.0 mg/L is thought to be the hazardous threshold value for cyanide in blood, as the fatal threshold varies from 2.5 to 3.0 mg/L (around 120 µM) [45]. The range of 500 to 3500 µg/kg of body weight is considered the acute fatal oral dose of cyanide for humans. Ingesting 20 µg/kg CN daily may cause acute symptoms, while 80 µg/kg CN can cause persistent symptoms [59,70]. To prevent human poisoning, the Food and Agriculture Organization (FAO)/World Health Organization (WHO) has established a limit of 10 mg HCN/kg dry weight in edible plants, which must be followed with caution during processing due to the heat sensitivity of cyanogenic glycosides [71].

Goitrogens, present in many plants, including cruciferous vegetables and soy, are known to inhibit iodine utilization and the synthesis of thyroid hormones, thus leading to hypothyroidism and goiter [72]. One study from the Olsztyn province in Poland found a 1.5-fold higher risk of thyroid cancer associated with regular consumption of cruciferous vegetables [73]. Another study concluded that the high prevalence of thyroid cancer among Melanesian women in New Caledonia is due to a combination of a high intake of cruciferous vegetables and mild iodine deficiency [74]. However, the data on the whole appears controversial and inconclusive, suggesting the conclusion that in any relatively balanced diet, goitrogens are not likely to cause serious harm [2].

Phytoestrogens have been associated with endocrine disruption and a higher risk of estrogen-sensitive cancers, but also with the alleviation of menopausal symptoms, obesity, and diabetes, among other things [75]. An enormous amount of research has been done on the health effects of phytoestrogens, but the only conclusion to be drawn from it is that much more is necessary. Both beneficial and adverse effects remain poorly understood and highly contentious [76].

Saponins are non-volatile, surface-active secondary metabolites found in over 100 plant species, including two major *Magnoliophyta* classes: *Magnoliopsida* and *Liliopsida*, as well as certain marine sources [77]. Triterpenoid saponins are present mainly in legumes such as alfalfa (*Medicago sativa*), chickpeas (*C. arietinum*), broad beans (*Vicia faba*), soybean (*Glycine max*), lentils (*Lens culinaris*), kidney beans (*Phaseolus vulgaris*), peanuts (*Arachis hypogaea*), but also in sunflower seeds (*Helianthus annuus*), ginseng roots (*Panax quinquefolius*), horse chestnut (*Aesculus hippocastanum*), tea leaves (*Camellia sinensis*), licorice roots (*Glycyrrhiza glabra*), quillaja bark (*Quillaja saponaria*), spinach leaves (*Spinacia oleracea*), quinoa seeds (*Chenopodium quinoa*), and sugar beets (*Beta vulgaris*) [23,60,61,78]. Steroidal saponins mostly accumulate in crop plants such as yams (*Dioscorea* spp.), alliums (*Allium* spp.), asparagus (*Asparagus officinalis*), fenugreek (*Trigonella foenum-graecum*), yucca (*Yucca* spp.), ginseng (*P. quinquefolius*), aubergine (*Solanum melongena*), tomato (*Solanum lycopersicum*), and capsicum peppers (*Capsicum annuum*) [78,79].

Since both types of saponins contain hydrophilic glycoside moiety and lipophilic aglycon, they are amphiphilic. These compounds are therefore highly valued in the food, cosmetic, and pharmaceutical industries as natural emulsifiers, foaming, stabilizing, and drug delivery agents [60,80,81]. In contrast to cyanogenic glycosides, saponins exhibit advantageous biological activities, including antioxidant, anti-diabetic, anti-inflammatory, hepatoprotective, immunomodulatory, anticancer, and antimicrobial effects [72,82,83,84,85]. They can also prevent cardiovascular issues by lowering blood levels of “bad” cholesterol [61]. In light of their numerous health advantages, the primary factors restricting the nutritional intake of saponins are their extremely bitter taste, irritating effect on the throat, low physiological availability, negative impact on protein, lipid, and mineral absorption in the body, and hemolytic properties [1,61,85]. However, it is worth noting that these effects can greatly vary between saponins depending on the chemical structure of their aglycones and the glucydic composition [86]. Thus, some saponin-rich plant products, such as licorice roots, yucca, and *Quillaia* extract, have been approved as food additives by the FDA [61,87].

Saponins are popular tumor-suppressing agents, apparently able to induce apoptosis and cell cycle arrest in tumor cells [85]. However, there is some experimental evidence that they also are potent hemolytic agents, promoting apoptosis of human erythrocytes (eryptosis) in addition to their lysis by more conventional ways such as increased Ca^2+^ influx and ceramide formation [88,89]. There is scant information on the mechanism by which they may limit the bioavailability of nutrients (i.e., act as antinutrients).

### 2.3. Phenols

**Figure 2:**
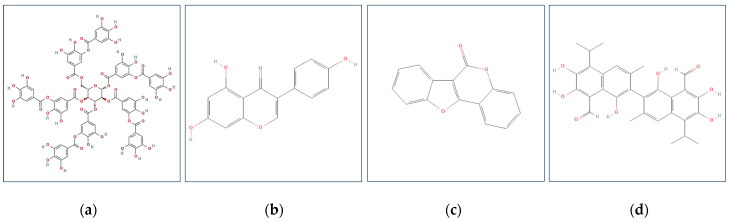
Chemical structures of phenolic antinutrient compounds in plant food. Examples: (**a**) Gallotannin; (**b**) genistein; (**c**) coumestan; (**d**) gossypone.

Phenolic antinutrients in plants are (i) tannins, stringent polyphenolic compounds usually divided into condensed (also known as catechins, condensed flavonoids) and hydrolyzable (gallo- or ellagitannins, depending on whether gallic or ellagic acid is the base unit) [90]; (ii) phytoestrogens, which may be divided into four classes of phenolic compounds: isoflavones (genistein, daidzein, glycitein, and biochanin A), lignans, stilbenes, and coumestans [91]; (iii) gossypol, a yellowish polyphenolic compound present in all parts of the cotton plant (*Gossypium* spp.) but especially abundant in cotton seeds (up to 2.4%) [92]. The structural formulae of several tannin and phytoestrogen compounds are shown in Figure 2.

Tannins are polyphenolic secondary metabolites found in almost every part of higher plants, such as the bark, wood, leaves, fruit, roots, seeds, and plant galls [1,93]. Tannins confer bitterness and astringency of taste that may be considered a sign of high quality in red wines, fruit juices, and beer [94,95]. The increased tannin production is frequently associated with plants’ defense responses against infection, insects, or herbivores [96,97,98].

Recently, plant tannins have attracted attention because of their multifunctional properties beneficial to human health, such as antioxidants [99,100]. However, excessive consumption of large amounts of tannins has been reported to cause side effects or even have a negative health impact [101], which depends on the chemical structure, dosage, and daily intake tolerance [102]. Hydrolyzable tannins (gallotannins and ellagitannins) are easily hydrolyzed by acids, bases, or enzymes into carbohydrate and phenolic acids. Sources rich in hydrolyzable tannins are apricots, peaches, nuts, mangos, cloves, pomegranates, plums, berries, teas, grapes, and guavas [103]. Condensed tannins (proanthocyanidins) are polymer products of flavan-3-ol (catechin, epicatechin), flavan-3,4-diol, or a mixture of these [104]. They are resistant to hydrolysis and are present in commonly consumed food products like legumes, seeds, beans, lentils, peas, millet, sorghum, and fruits (pears, apples, cranberries) (Table 2).

Tannins reportedly harm iron absorption and iron stores. However, while studies in pig and rat models have shown a significant reduction of hemoglobin levels and hepatic iron, others have failed to do so. Nor have human studies managed to redress the balance [105]. One study conducted with 11 women found that long-term consumption of condensed tannins (up to 1.5 g three times a day for 3 weeks) caused no change in iron bioavailability [106]. A curious study of 55 female students from Indonesia found that 71% of them regularly drank tea, and of these, almost 54% had hemoglobin levels lower than usual. In contrast, nearly 64% of those who did not drink tea had normal hemoglobin levels. The difference is attributed to the high levels of tannins in tea [107]. Cows fed with a diet based on sorghum showed decreased milk production in a dose-dependent manner with the addition of tannic acid up to 320 g/day [108]. A study among 64 stunted and overweight adolescents from Semarang City, Indonesia, found a significant correlation between high intake of tannins and abnormally low serum levels of hemoglobin and transferrin receptors. Interestingly, the correlation was not significant in the case of high phytate intake [109].

### 2.4. Anti-Minerals: Phytates and Oxalates

Phytates and oxalates are salts of the phytic and oxalic acids, respectively. They are strong chelators of metal cations (Zn^2+^, Mg^2+^, Ca^2+^, and Fe^2+^), thus prone to limiting their bioavailability [110].

#### 2.4.1. Phytate

**Figure 3:**
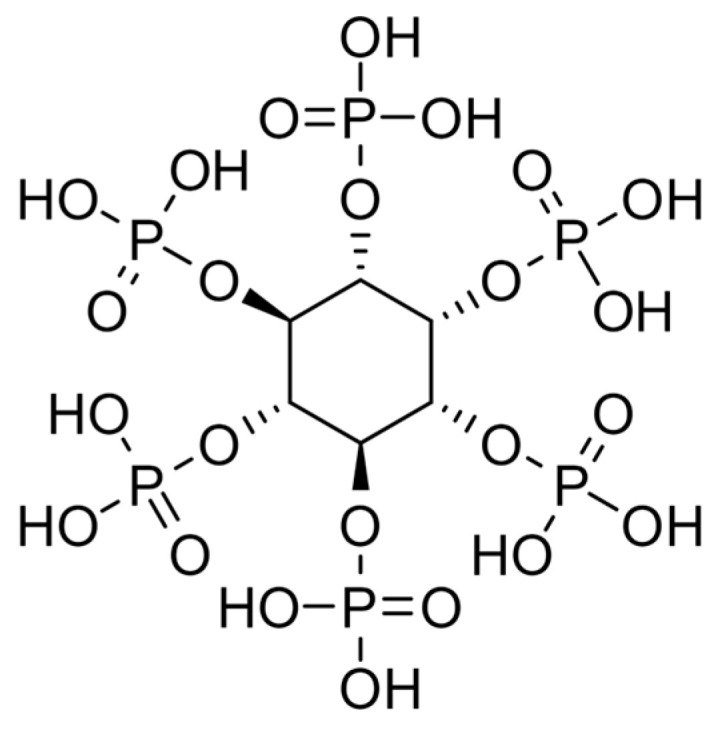
The structural formula of phytic acid.

Phytate, as a salt complex with myo-inositol, is presented in various plant-based foods, including seeds, grains, nuts, and legumes. Phytic acid is the major phosphorus storage in numerous plants and soil [111]. In general, seeds such as grain legumes, cereals, and oilseeds tend to contain large amounts of phytate, providing phosphorus for germination. Roots, tubers, and fruits typically contain moderate amounts of phytate; the lowest level is in leaves [112]. Phytate is a six-carbon cyclic structure called inositol, with phosphate groups esterified at various positions on the inositol ring (Figure 3). This structure has multiple negatively charged positions, allowing interaction with positively charged ions. Thus, phytates impair negatively the absorption of many metal ions, most notably Zn^2+^, Fe^2+^/^3+^, Ca^2+^, Mg^2+^, Mn^2+^, and Cu^2+^. As a result, the non-soluble phytate–mineral complexes formed cannot be absorbed by the digestive tract. Furthermore, phytic acid can also chelate with amino group derivatives in proteins, reducing their bioavailability and absorption [113].

Human studies indicate that phytates impair magnesium and iron absorption [114,115]. Consuming 4–9 mg of phytic acid per 100 g of food can lead to a five-fold decrease in human iron absorption. However, the latter study found that iron absorption is additionally inhibited by polyphenolic compounds, like the phytates, in common beans (*Phaseolus vulgaris*). Dephythinization alone did not increase the iron absorption, but when the polyphenols were removed too, there was a 3.4-fold improvement. This synergistic effect is relatively common and should be considered when studying the combined ANF effects. A diet high in phytates has been linked to nutritional disorders like rickets and osteomalacia (“bone softening”) in both children and adults [116]. Lonnerdal et al. [117] found that the inhibitory effect of phytate on calcium and zinc absorption depends on the degree of phosphorylation of inositol. At higher degrees of phosphorylation, such as inositol with five or six phosphates (InsP5, InsP6), there is a significant inhibition of calcium and zinc absorption. However, at lesser degrees of phosphorylation, no such effect is observed. The number of phosphate groups attached to inositol plays a crucial role in determining the inhibitory effects of phytate on mineral absorption. Processed foods may contain significant amounts of inositol phosphates with fewer than six phosphates. During fermentation, microbial enzymes break down phytate into its lower phosphorylated forms, such as inositol triphosphate or inositol tetraphosphate. This breakdown can occur through other food processing techniques [118]. On the other hand, several recent studies demonstrated strong proof of the anti-cancer activity of phytic acid [119], its potential in the alleviation of diabetes symptoms [120], vascular calcification prevention [121], potential protective effect against neurodegenerative Parkinson’s disease [122], prevention of dental caries and kidney stones, and as a possible antidote to acute lead poisoning [102].

#### 2.4.2. Oxalates

Oxalic acid (ethanedioic acid, C_2_H_2_O_4_) is a compound found in various plant foods, which forms soluble or insoluble salts known as oxalates. Oxalates are commonly found in foods such as cruciferous vegetables, and leafy greens like spinach, kale, broccoli, soybeans, nuts, berries, beans, and certain spices are also known to be rich sources of oxalates. Cooking these foods can help reduce their oxalate content, making the minerals they contain more available for absorption [123].

The oxalate ion is negatively charged, and it can form salts with different positively charged ions, like K^+^, Na^+^, Ca^2+^, Mg^2+^, Zn^2+^, and Fe^2+^ [124]. The solubility of the different salts varies—for example, sodium and potassium oxalates are soluble, while calcium, magnesium, zinc, and iron salts are less soluble to practically insoluble oxalate compounds. The insoluble calcium oxalate forms indigestible crystals and thus reduces the amount of calcium available for absorption by the body. Over time, high intake of oxalate-rich foods can contribute to the formation of kidney stones, as these crystals can accumulate and potentially cause health issues like a condition known as nephrolithiasis [125,126,127,128]. Oxalate homeostasis is critical for the prevention of kidney failure. Superfluous oxalates, dietary or endogenous, promote chronic kidney disease (CKD) in a more complex and still poorly understood way than the formation of kidney stones. Moreover, elevated plasma or urinary levels of oxalates have been linked with many other pathological conditions besides CKD, among them diabetes, atherosclerosis, obesity, and even neurological disorders [124]. Pairing oxalate-rich foods with calcium-rich foods can help mitigate the effects of oxalates on mineral absorption [129,130]. It has been observed that the risk of kidney stone formation is higher among individuals with lower dietary calcium intake. Adequate calcium intake helps bind dietary oxalate in the gut, reducing oxalate absorption and, consequently, the risk of kidney stone formation [131].

In contrast to other antinutrients, such as phytates and tannins, which have been studied for their potential health benefits (anticancer properties, improved glucose metabolism, antioxidant effects), oxalates primarily pose a health concern [128]. Oxalate poisoning in domestic animals has a rich history, with ruminants showing greater resistance to high oxalate levels due to oxalate-degrading bacteria in the rumen. Oxalate toxicity levels have been widely estimated in various studies—between 450 mg/100 g and 5 g/100 g food [132,133]; however, the critical levels of soluble oxalates remain a matter of debate, and more long-term studies are needed to establish them [134]. Dysfunctional oxalate metabolism in a mouse model has been described as one of the driving forces, and possible therapeutic targets, of atherosclerosis. Mice with knockout genes for alanine-glyoxylate aminotransferase (AGXT) showed decreased glycine/oxalate ratio and increased atherosclerosis, a striking effect confirmed in human patients. In isolated macrophages, oxalate-induced oxidative stress and mitochondrial dysfunction appear to be contributing factors [135]. However, it is important to note that plant foods that contain oxalates also provide numerous bioactive compounds that offer significant health benefits. Despite the presence of oxalates, the consumption of these nutrient-dense foods is generally encouraged due to their overall health benefits.

A summary of the effects of antinutrients on human health is presented in Table 3.

### 2.5. Anti-Vitamins and Anti-Enzymes

Anti-vitamins are molecules that inhibit the actions or absorption of vitamins, thus diminishing or abolishing their specific functions. Historically, the first example of an anti-vitamin is phytic acid, which was found to be the antagonist of vitamin D. Then, thiaminases of raw fish, shellfish, Brussel sprouts, and red cabbage have been found to induce irreversible degradation of the target vitamin B1 (thiamine), causing beriberi symptoms [136]. Anti-vitamin B1 was also found in plants such as bracken fern (Pteridium aquilinum), one of the five most vascular plants in the world [137].

Another anti-vitamin example is the anti-pyridoxine factor, which concurs with vitamin B6 (pyridoxine). It is abundant in linseed and comprises amino acid D-proline (that occurs as peptide linatine) combined with glutamic acid. L-amino-D-proline is about 4 times more active than linatine, which is known as a strong antimicrobial agent [137].

Avidin, which hampers biotin absorption, is especially abundant in soybeans (rich in anti-vitamin A, D, and E factors) and eggs white. Avidin is an antinutrient that binds vitamin B12, exhibiting the highest known ligand–protein affinity in nature [138]. The bioavailability of biotin to consumers can be additionally compromised by the tight complex between avidin and its associated vitamin B8 (inositol). Anti-vitamin E, isolated from soya protein, probably acts as tocopherol oxidase [137].

Vitamin K complex is a fat-soluble vitamin comprising of the following substances: phylloquinone (K1-predominant), dihydro phylloquinone (dK), menaquinone (K2), and menadione (K3). The poor gastrointestinal absorption of vitamin K is affected by plant ANF coumarin (a heterocyclic class of organic compounds), a substance found in grapefruits, alfalfa, and capers. Its consumption delays blood clotting if consumed in high doses [139,140].

Anti-enzymes are, for instance, the cholinesterase inhibitor solanine in plants from the genus *Solanum* (potatoes, tomatoes, eggplant), an arginase inhibitor in sunflower seeds, and amylase inhibitors common wheat, oats, and rye [141,142].

Well-known protease inhibitors in eggs are ovostatin, ovomucoid, ovoinhibitor, and cystatin. They inhibit digestive enzymes, including pepsin, trypsin, and chymotrypsin, and hamper digestion in the human gastrointestinal tract [138]. Molecules such as ovomucoid and cystatin contain multiple disulfide bonds and are resistant to gastric juice and proteases in the digestive system. However, since they are susceptible to thermal denaturation, they can be easily destroyed during cooking.

## 3. Non-Microbial Methods for Antinutrients Detoxification

Traditional ethnic food processing methods and technologies such as soaking, milling, roasting, cooking, and sprouting reduce anti-nutritional components in foods. Milling flour is the oldest method of removing the bran layer from grains, thus reducing the content of phytic acid, lectins, and tannins. Experimental data revealed that the milling and heating process during the preparation of chapati from pearl millet significantly reduced phytic acid and polyphenols and increased the quantity of digestible starch and protein [143]. The study of Udensi et al. [144] indicated that 6 h of soaking reduced phytic acid by 27.9% at room temperature in Mucana flagellipes, and 24 h of soaking reduced it by 36.0%. Wheat and barley require soaking from 12 to 24 h, which activates endogenous or exogenous phytase enzymes that can improve the in vitro solubility of minerals such as zinc and iron by up to 23% [145]. Soaking reduces phytate content more successfully if carried out at 45 °C and 65 °C and for a long time; for example, phytic acid concentration in chickpeas decreased by 47.45 to 55.71% when the soaking time was increased from 2 to 12 h. Moreover, the soaking reduces cooking time, enhances the release of endogenous phytases, and provides essential moisture for germination. On the other hand, conventional heat treatment reduces the phytic acid content by only 5 to 15%. When pressure is involved in the process, as in autoclaving, detoxification is more rapid and decreases much of the ANF content. For instance, autoclaving was applied to diminish the oxalate content in taro leaves (47% reduction) and for soybean trypsin inhibitor activity decrease [23]. Nakitto et al. [146] reported a high reduction in tannin and phytic acid contents in beans, which was greater in steamed beans (31.1 and 46.1%, respectively) as compared to roasted beans (4.25 and 18.42%, respectively).

Several studies have compared soaking and fermentation as ANF detoxification methods. Ndouyang et al. [147] compared soaking (for 72 h) and fermentation (for 48 h, by unspecified microorganisms) of *Tacca leontopetaloides* tubers. Phytates were reduced by both methods, but only by about 20%. Alkaloids and saponins were significantly reduced by both methods, 43% by soaking and 51 to 77%, respectively, by fermentation. Soluble oxalates were reduced by almost 94% by fermentation yet remained virtually unchanged by soaking. Flavonoids and hydrolyzable tannins increased by fermentation by 52% and 99%, respectively. Comparing both methods to detoxify bamboo shoots, Chongtham et al. [148] showed that cyanides were reduced by fermentation (to 23%) but not by soaking (remaining 43%). Condensed tannins and insoluble oxalates were increased by both methods, but even in this case fermentation seems preferable because of the lesser extent, 59% and 22% against 101% and 352%, respectively [149].

Soaking for 16 h in distilled water and, even more so, cooking for 60 min on a hot plate reduced the levels of tannin, phytate, and oxalate in red, white, and black kidney beans (*Phaseolus vulgaris*) from Ethiopia. However, there was a correlation with the bioavailability of calcium and iron. Only zinc showed increased levels in all three types of beans, and only after soaking. The cooking decreased or did not change significantly the zinc levels. Similar studies from India, Pakistan, Nigeria, and Kenya produced dissimilar results [150]. One study has revealed lower levels of condensed tannins in spontaneously fermented-dried and blanched-dried African leafy vegetables (ALV) from sub-Saharan Africa compared to cooked samples. The bioavailability of zinc and iron was notably increased by both drying techniques in three out of five ALVs, the exceptions being Ethiopian kale and amaranth [151]. It has to be noted that during soaking diffusion, large amounts of salts also leave the food, while vitamins are degraded during cooking. Compared to these methods, fermentation achieves the same or better levels of detoxification along with the following advantages: the process is performed under mild conditions (without high temperatures or pressure), relatively faster than germination, without loss of vitamins and salts, and with improved taste and nutritional qualities of food. However, recently developed innovative non-microbial approaches to remove ANFs are also promising. Such examples are extrusion [152,153,154], dielectric heating treatments, such as radiofrequency and microwave radiation [153,155], and ultrasound technology, the last able to decrease phytate and tannin content in finger millet by 66.98 and 62.83%, respectively [156]. Cold plasma treatment is particularly promising since it substantially reduces the phytic acid content, and trypsin inhibitor activity [157,158,159]. The last ANF can also be removed by methods for optimized protein extraction [160]. Methods for genetic manipulation of plants aimed at reducing the synthesis of antinutrients are also being developed. Breeding strategies to alter the anti-nutritional components for enhanced bioavailability of nutrients in foods are applied [161]. Selection of low-ANF-producing genotypes of plants, followed by fermentation, is the most applicable [162].

## 4. Bacterial Reduction of Antinutrients

Fermentation provides an alternative to the physical method of antinutrient detoxification that is neither more expensive nor more difficult. While fermentation is more time-consuming, it may have the additional benefit of possible probiotic properties of the bacterial strains involved. A number of studies involving lactic acid (LA) fermentation of sauerkraut, pickled cucumbers, or fermented soya products have reported decreased content of oxalates, tannins, and glucosinolates. LA fermentation is the best approach to diminishing the adverse effects of phytate-rich cereals (pearl millet, maize), and pseudocereals [9,20]. In addition, fermented foods often have superior health-promoting effects, for instance, antioxidant potential. The case of each antinutrient must be considered individually within the context of its food source and the fermentation conditions [163]. Examples of ANF degradation during fermentation processes and the reduction levels are presented in Table 4.

Enzymatic degradation appears to be the major mechanism of microbial reduction of antinutrients. A genomic study of 351 bacterial genomes found that ANF-degrading enzymes are relatively rare. Only 22 *Lactobacillus* strains contained between 6 and 13 genes for metabolizing ANFs. Five of them could act on six ANFs and 12 on five ANFs. *Bacillus* strains showed wider distribution but more limited application of ANF-degrading genes: two strains had seven of them, and 31 had six. None of these 33 strains, however, was effective against more than 4 ANFs [5].

Some of the most common ANF-degrading enzymes are hydrolases and include tannase and phytase. Tannase hydrolyzes ester and depside bonds in tannins, which leads to gallic/ellagic acid and glucose. Gallic acid can be further metabolized to pyrogallol by gallate decarboxylase in *Lp. plantarum*. Phytase splits phytates into inorganic phosphates and lower esters of myoinositol, or even free myoinositol in some cases. This is usually accompanied by increased levels of minerals, especially Ca^2+^ and Mg^2+^. Oxalates are something of an exception. They are decarboxylated to CO_2_ and formate, which can be used as a source of carbon and energy by some lactobacilli such as *Lp. plantarum* and *L. acidophilus* [173]. The most important bacterial hydrolases regarding antinutrients are β-glucosidases. A large class of diverse enzymes common in lactic acid bacteria and bifidobacteria, β-glucosidases can utilize a vast variety of substrates with downstream effects that may or may not be desirable. When they split the sugar side chains of saponins, β-glucosidases have a beneficial effect, lowering the solubility of saponins and reducing their harmful effects. But when they hydrolyze cyanogenic glycosides, β-glucosidases promote the generation of cyanide, an inhibitor of the cytochrome C oxidase (Complex IV) from the mitochondrial respiratory chains [53,173].

### 4.1. Microbial Degradation of Lectins

Lectins are highly unstable under high temperatures. A study of black turtle beans found non-detectable levels of hemagglutinating activity after 20 min at 80 °C, and that with the very respectable 1.64 × 10^6^ HU/mg to begin with [174]. Thermal instability might be an explanation for why relatively little work has been done on bacterial degradation of lectins, and why some of it is in domestic animals. It has been known for decades that lectins are bound and degraded in vitro by rumen liquor to various degrees, but the exact mechanisms remain elusive [175,176,177,178]. Unspecified starter culture achieved a 98% reduction in the lectin content in *Lens culinaris* (79 g/L) within 96 h. The natural microbiota of lentils, in this case var. Magda 20 from Albacete (Spain), is presumably responsible for that effect and therefore worth future study [179]. Solid-phase fermentation of soybean meal by strains of *Aspergillus* spp. and *Bacillus* spp. resulted in a significant reduction in lectin levels and increased organic acid production, especially lactic acid.

### 4.2. Microbial Degradation of Cyanogenic Glycosides

Cyanogenic glycosides have a complex fate in the intestinal environment or during fermentation. Generally, they are hydrolyzed by bacterial β-glucosidases to cyanohydrins (hydroxynitriles) and glucose, or gentiobiose. Hydroxynitrile lyase catalyzes further conversion to HCN and the respective aldehyde or ketone, but cyanohydrins are unstable and often degrade spontaneously under physiological conditions (pH > 5, t > 35 °C).

**Figure 4:**
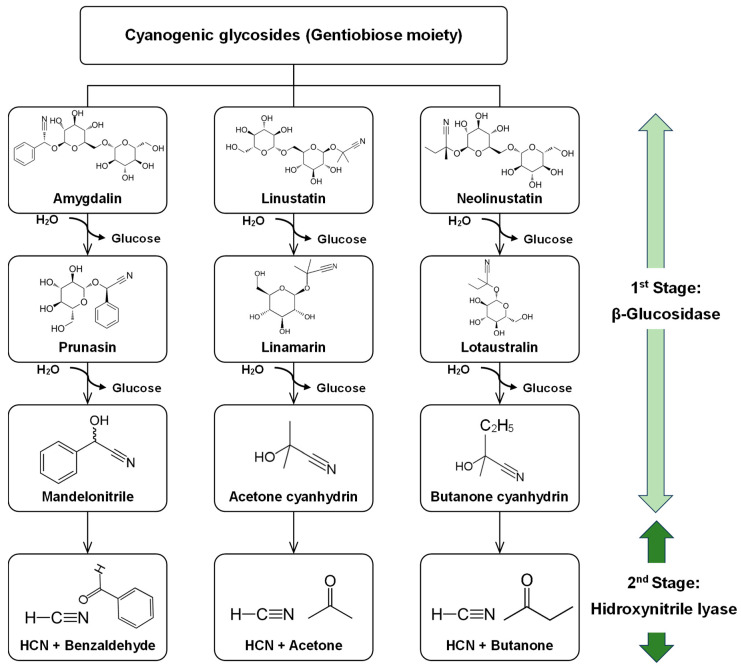
Two-stage hydrolysis of gentiobiose containing cyanogenic glycosides. Structural formulae were provided by the public domain https://pubchem.ncbi.nlm.nih.gov/ (accessed on 6 May 2024).

The rate of cyanide production depends on several factors, among them the nature of the sugar moiety. Glycosides that contain gentiobiose undergo a two-stage hydrolysis until the respective cyanohydrine is formed (Figure 4).

Cyanohydrins differ in their speed of spontaneous degradation. Hydrolysis of prunasin, for instance, results in much more rapid cyanide production than the hydrolysis of linamarin [53,180,181]. There are several ways to dispose of the cyanide that results from the hydrolysis of cyanogenic glycosides. Possibly the most widespread is to convert it to the much less toxic thiocyanate. The enzyme rhodanese (EC 2.8.1.1, thiosulfate: CN sulfurtransferase) is mainly responsible for this. *Enterococcus faecium* and *E. gallinarum* isolated from rumen showed high rhodanese activity, ranging between 4.42 and 25.49 mg hydrogen CN equivalent/L, but they were the exception rather than the rule. Only 6 from a total of 44 strains isolated from the rumen of domestic buffalo, dairy cattle, and beef cattle showed rhodanese activity [182]. Rhodanese is by no means the only microbial way of dealing with cyanide; there are plenty of others, but they are more relevant to the bioremediation of industrial wastes than to food fermentation [101].

A recent study of *Lactobacillus* genomes revealed that both homofermentative and heterofermentative species have a large genetic potential for the synthesis of β-glucosidases. According to Dymarska et al. [183], the genomes of *Lp. plantarum*, *Lacticaseibacillus paracasei*, *L. crispatus*, and *Companilactobacillus paralimentarius* encoded for 8 to 22 enzymes, predominantly phospho-β-glucosidases, with predicted activity on β-glucosides [183]. Both species, *Levilactobacillus hammesii* and *Furfurilactobacillus milii,* were reported to encode for three β-glucosidases, while *Furfurilactobacillus rossiae* contains a gene for one. Cultivation experiments with amygdalin, esculin, salicin, quercetin, genistein, and ginsenosides prove that the genetic profile of the strains matches the acting on hydrolysis of β-glucoside enzymes.

*B. subtilis* KM05 with a considerable ability to utilize cyanogenic substrates in cassava flour led to the isolation of the enzyme linamarase, a β-glucosidase of 53 kDa with considerable ability (9.6 U/mL) to degrade linamarin, which accounts for 80% of the cyanide content in cassava. Limanarase may prove a useful additive during the processing of fresh cassava roots, which includes a long process of washing and milling during which most of the HCN is removed anyway [184]. Purified limanarase cannot be utilized in fermented foods. The *Bacillus* strain from which it was isolated is another matter, but it must be investigated what mechanisms (if any) of cyanide disposal are available in the said strain. *Bifidobacterium*, a genus of high importance in both human gut microbiota and food fermentation, has shown a remarkable ability to utilize plant glycosides, including cyanogenic ones. An ambitious study of 115 strains from eight different species showed widespread β-glucosidase activity. Amygdalin, the most common cyanogenic glycoside in almonds, was the preferred substrate: 54% of the strains were able to use it as a sole carbon source. Strains of *Lp. plantarum* and *Lp. paraplantarum* degraded amygdalin, as reported by Menon et al. [185], and Lei et al. [186]. *L. acidophilus* MTCC-10307 achieved 66% cyanide reduction (from 5.17 mg/g initial concentration) in a fermented beverage prepared from linseed (*Linum usitatissimum*). The reduction was obtained after only two days of fermentation and, while comparable to other LAB isolates, was markedly superior to a couple of *Saccharomyces* spp., which managed only 30%. Interestingly, roasting linseed led to an insignificant reduction in the cyanide content—a mere 8% [186]. Cyanide reduction from 72.72 to 5.18 mg/kg (83%) in “mchuchume”, a fermented cassava beverage from Tanzania, was achieved with *L. delbrueckii* at 30 °C, although it took nearly 72 h to reach the maximum effect [187].

### 4.3. Microbial Degradation of Saponins

Successful degradation of saponins has been achieved by several bacterial strains. *B. megaterium* O1, isolated from soapnut (*Sapindus saponaria*), proved capable of surviving on saponin extract from *Quillaja saponaria* as a sole carbon source. While the strain is not a contender in food fermentations, its genome has been sequenced and published and may yield novel insights into the mechanisms of saponin degradation [188]. Likewise, *Bif. longum* KACC 91563, a member of the human gut microbiota, has proved a good model organism for studying enzymatic activity. BIBG3, a putative GH3 β-glucosidase from *Bif. Longum,* was cloned and studied on functional and structural levels. It was found that the enzyme was highly efficient in hydrolyzing the saponins furostanol and ginsenoide Rb1, removing a glucose moiety that is bound in a deep pocket of three unique loops. At least two amino acids (R484 and H642) were found to be critical for the enzymatic activity [189].

*Lp. plantarum* 1 in quinoa dough, inoculated with 9 × 10^8^ CFU/mL at pH 3.73, achieved a 71% decrease in the saponin content after 4 days of fermentation (80.2 mg/100 g vs. initial concentration of 272.3 mg/100 g). The total sugar content more than quadrupled for the same time (614.9 mg/g vs. 153 mg/g initial concentration), partly due to the amylolytic activity that degrades starches in the food matrix, but also partly due to the desaponification process, during which sugar moieties are released from the aglycone part of saponins. In other words, saponin degradation improves the nutritional value of the final product, in this dehydrated base for quinoa soup [190]. A combination of *Str. thermophilus* 14085 and *Bif. infantis* 14603 in fermented soymilk for 24 h at 37 °C achieved a reduction in saponins between 32% and 47%, depending on what was used to obtain the extracts from soymilk, 80% methanol, 50% acetone, or water [191]. A starter culture for hemp (*Cannabis sativa* L.) sourdough containing *Lp. plantarum*, *Pediococcus acidilactici*, and *Leuc. mesenteroides* also decreased the saponin levels during fermentation [192].

Saponins in ginseng (*Panax* spp.), usually called ginsenosides, have received much attention because of their medicinal properties. *Lp. plantarum* CRNB22, among the 6 out of 28 strains with β-glucosidase activity isolated from Korean kimchi, showed a strong ability to hydrolyze saponin Rb1, one of the most common ginsenosides (together with Rb2, Rc, Re, and Rg1) in ginseng. Further study is needed to characterize better the hydrolysis products of Rg1, but the strain looks like a promising additive to functional foods rich in ginsenosides [193]. The same may be said of *Lentilactobacillus buchneri* URN103L, which demonstrated higher extracellular β-glucosidase activity than any strain on record, including the ability to hydrolyze Rb1. Whether the hydrolysis products, here designed Rd and Rg3, have increased beneficial effects, and just what these effects might be, remains a promising target for future research [194]. Pfam et al. [5] suggest that β-glucuronidase is the enzyme that breaks the β(1→4) glycosidic bond between the aglycone and sugar residues of tetrose in tea seed saponins. Such endo-β-glucuronidase activity has been demonstrated for *L. crustorum* capable of reducing the hemolytic activity of tea seed saponins in vitro [195]. A search for β-glucuronidase genes in bacterial genomes revealed that they were present in strains of *Lactobacillus* species, but not in *Pediococcus* and *B. subtilis* genomes. Among 151 genomes, 31 genomes of *Lactobacillus* species contain at least one gene encoding β-glucuronidase. Notably, *L. hammesii* DSM 16381, *L. parabrevis* LMG 11984, and *L. secaliphilus* strain DSM 17896 contained four genes encoding putative β-glucuronidase (NCBI Genbank accession numbers NZ_AZFS01000047, NZ_JQCI01000099, and NZ_JQBW01000005, respectively). These findings indicate that the best genetic profile suggesting a reduction in the hemolytic activity of saponins possesses the probiotics of the genus *Lactobacillus* [5]. Root microbiome has also been demonstrated to possess a remarkable ability to degrade saponins (>80%) [194]. Unfortunately, methanogenic archaea are quite inapplicable in food fermentations.

### 4.4. Microbial Degradation of Tannins

**Figure 5:**
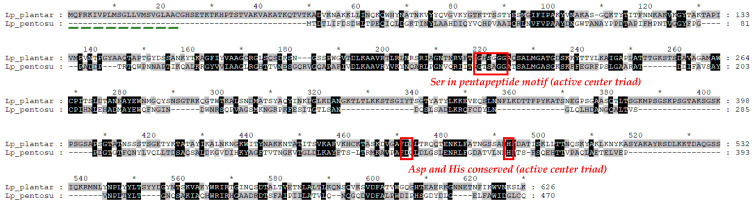
Protein sequences alignment of tannase of *Lp. plantarum* (KRL35904) and *Lp. pentosus* (QAB08722) using the free software GeneDoc v2.7 [197]. Identical residues are shown in black; the non-identical—are in grey and white; dashes indicate gaps introduced to maximize similarities; the asterisk means consecutive counting of ten amino acids. The Serine residue participating in the conserved amino acid motif Gly-X-Ser-X-Gly), and Asp and His of the active center triad are shown in red boxes; the putative signal peptide of *Lp. plantarum* tannase is green-dashed underlined.

Tannases (tannin acyl hydrolases, E.C. 3.1.1.20) are common in bacteria, yeast, and fungi, varied in sequence and structure but sharing the common pentapeptide Gly-X-Ser-X-Gly typical for serine hydrolases [195]. Tannases have been studied extensively in terms of their hydrolytic activity and industrial potential, but relatively little is known about them on genetic and molecular levels. One classification divides them into two subtypes depending on sequence similarity and the presence (subtype B) or absence (subtype A) of an Asp residue (Asp-419) in the catalytic triad [196] (Figure 5).

Structural studies of novel tannases TanALb and TanBFnn, isolated from *Lachnospiraceae* bacterium in the ruminant gastrointestinal tract [198] and *Fusobacterium nucleatum* [199], respectively, have revealed the existence of a flap domain critical for substrate recognition and specificity. Since *F. nucleatum* is associated with the pathogenesis of colorectal cancer (CRC), inhibition of TanBFnn, accompanied by increased levels of gallotannins, may decrease the survival of *F. nucleatum* and possibly even the risk of CRC. TanALb was able to hydrolyze tannic acid and, to a lesser extent, four esters of gallic acid with alcohols of various lengths (methyl, ethyl, propyl, and even the C12 lauryl) [200].

Application of tannases in functional foods has lagged behind their structural studies, although the crystal structure of *Lp. plantarum* tannase was solved a decade ago [201]. The genome of *Lp. plantarum* encodes three tannases: a widely spread intracellular tannase (TanB), a strain-specific intracellular broad esterase, and an extracellular tannase (TanA), the latter only described in the strain ATCC 14917. Extracellular tannases in bacteria are quite rare; therefore, the genetic techniques for their detection are preferred. Among 115 screened LAB strains (77 of them, *Lp. plantarum*), Pulido-Mateos et al. (2022) found eight tannin-transforming *Lp. plantarum* strains, which contain the *tanA* gene, and six of them presented TanA activity [202].

From 117 *Bacillus* strains isolated from Miang, a fermented tea from Northern Thailand, 95 (81% of all) proved to be tannin-tolerant, but only in 21 of them (22% of the tannin-tolerant) was extracellular tannase activity detected. More than half of the strains were identified as *B. tequilensis* (11), but there were also *B. siamensis* (3), *B. megaterium* (3), *B. aryabhattai* (3), and *B. toyonensis* (1). All strains showed resistance to 0.3% bile salt, tolerance to highly acidic conditions (pH 3), and sensitivity to antibiotics. *B. tequilensis* K19.3, *B. tequilensis* K34.2, and *B. siamensis* K19.1 were deemed as particularly promising probiotics [203].

Among 155 analyzed genomes of *Lactobacillaceae* representatives, 17 genomes harbor a gene encoding for tannase; the genome of *Lp. plantarum* ATCC 14917 bears two [5]. An examination of the genome of a *Lp. pentosus* strain IG1, isolated from Spanish-style green olive fermentation, revealed the presence of several potential phenolic compound-degrading genes as deduced by their DNA and protein sequence homology [185]. Later, Carrasco et al. [204], after gene expression assay, showed that genes potentially coding tannase, gallate decarboxylase, and *p*-coumaric acid decarboxylase significantly increased their expression upon exposure to phenolic compounds.

Kanpiengjai et al. [205] characterized a new alkaline and thermostable tannase of *Lp. pentosus* strain BA-7. In Figure 5, the deduced amino acid sequence of the enzyme is compared to that of *Lp. plantarum*. The alignment shows the presence of all conserved regions in the enzymes, but also the possible presence of a signal peptide in *Lp. plantarum* tannase (a lipoprotein signal peptide with a cleavage site between positions 22 and 23).

### 4.5. Microbial Inactivation of Trypsin Inhibitors

Inactivation strategies for trypsin inhibitors range from traditional methods like heating, soaking, and germination to techniques like gamma and infrared radiation, high hydrostatic pressure, ultrasound, and ultrafiltration. Fermentation acquits itself with distinction in this select company. Its only notable drawback is that it is time-consuming. But compared to the rest, it is energy-saving, simple to perform, and, most important of all, benign towards vitamins, amino acids, and minerals [206,207,208].

Solid-state fermentation of soybean meal with *Lactobacillus brevis* CICC 6004 achieved 57.1% degradation of trypsin inhibitors at an initial concentration of 6.4 mg/g dry weight within 72 h [209]. Amylase inhibitors were reduced by 50.8% after 24 h fermentation of pearl millet, a staple food in the arid parts of Africa and Asia, during preparation of the fermented lohoh bread [209].

Fermentation of maize flour with *Lp. plantarum* for 48 h produced higher protein content and improved protein digestibility in vitro compared to *Saccharomyces cerevisiae*. Co-culture yielded the highest reduction in phytates (66%), tannins (75%), and trypsin inhibitors (64%) [210]. The amount of ATI (α-amylase/trypsin inhibitors), pernicious substances involved in the pathology of celiac disease and non-celiac sensitivity, was considerably reduced (>70%) in sourdough fermented with *Fructilactobacillus sanfranciscensis* DSM20451^T^ at pH 4.0. The result was comparable to wheat sourdough fermented with a multi-species culture, but superior fermentation with yeast and even baking (58–64% ATI reduction) [17].

### 4.6. Microbial Degradation of Phytates

**Figure 6:**
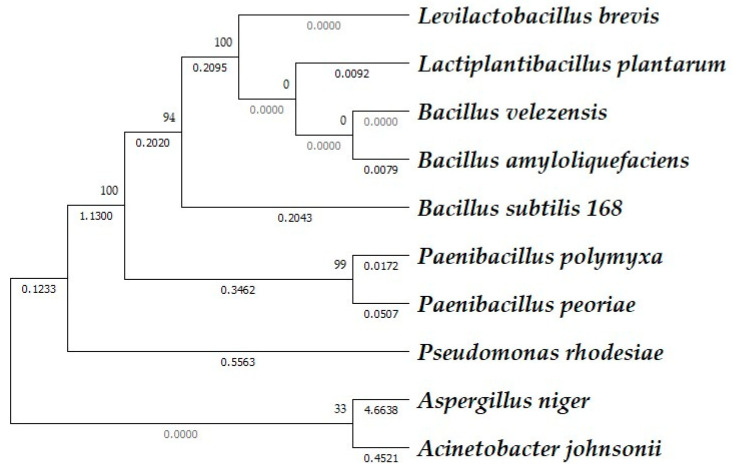
Amino acid alignments of microbial phytases: bootstrapped phylogenetic tree of phytase enzymes based on amino acid sequence comparison. Branches’ distances were computed using the maximum composite likelihood method; evolutionary analyses were conducted in the MEGA11 program [211].

Phytases belong to the group of phosphoric monoester hydrolases (EC 3.1.3.) and can be classified according to the number of phosphate groups released from the phytic acid: 3-phytase (EC 3.1.3.8), 4/6-phytase (EC 3.1.3.26), and 5-phytase (3.1.3.72). Alternative classification according to the structure of the catalytic domain divides phytases into cysteine phosphatases, histidine acid phosphatases, purple acid phosphatases, and β-propeller alkaline phytases (BPPs). The BPPs are typical for bacteria. The phylogenetic relatedness and similarity of phytases (with EC 3.1.3.8) of some microbial species (for which amino acid sequence data are available) are compared in Figure 6.

**Figure 7:**
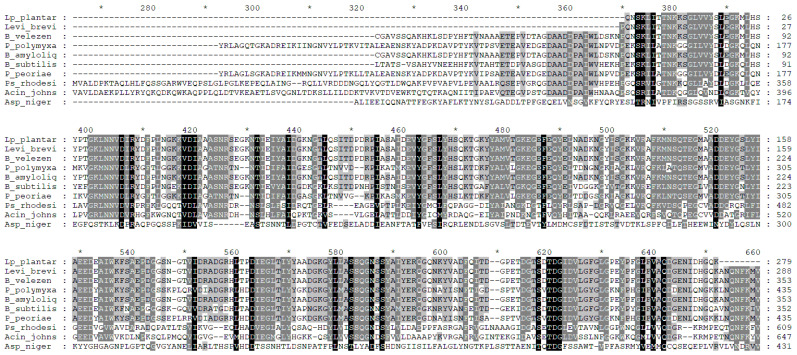
Demonstration of the conservative regions in microbial phytases, determined by protein sequence alignment using the free software GeneDoc [197]. The active center residues are shown in black; the semi-identical amino acid residues are in grey, the non-identical are white; dashes indicate gaps introduced to maximize similarities; the asterisk means consecutive counting of ten amino acids. In the comparison, the protein sequences with the following NCBI GenBank accession numbers were used: AVV65758.1 (*Lev. brevis*); AVV65757.1 (*Lp. plantarum*); CP103783.1 (*B. subtilis* 168); WP_325085020.1 (*B. amyloliquefaciens*); WP_130570879 (*B. velezensis*); CP024795.1 (*Paenibacillus polymyxa*); WP_348624070 (*P. peoriae*); WP_348943522.1 (*Acinetobacter johnsonii*); WP_348739472 (*Pseudomonas rhodesiae*); CAK38611.1 (*Aspergillus niger*).

Phytases of *Lp. plantarum* and *Lev. brevis* appear to be very similar to those of the *B. subtilis* group, e.g., *B. velezensis* and *B. amyloliquefaciens*. The amino acid sequences of the enzymes of *Paenibacillus* and *Pseudomonas* are quite phylogenetically distant from those of lactobacilli. Interestingly, *Acinetobacter johnsonii* phytase shows great similarity to fungal phytases (Figure 7).

Phytate-degrading bacteria have been used to improve cereal products obtained via dough fermentation. Of 150 LAB tested, *Enterococcus faecium* A86 (0.74 U/mL) and *Lp. plantarum* H5 (0.71 U/mL) showed the highest phytase activity [209]. *Fructilactobacillus sanfranciscensis* CCM 7699 and *Lp. plantarum* CCM 7039 after achieving 89% and 80% reduction in phytate content in tarhana (a mixture of wheat flour and yogurt) after 144 h of fermentation and initial amounts of 3.3–3.4 g/kg [182]. *Lentilactobacillus buchneri* MF58 decreased the phytate content by 59% during the fermentation of injera, a soft Ethiopian pancake prepared from tef flour [210].

The pan-genome analysis of Pham et al. [5], including 155 genomes of the *Lactobacillaceae* family, showed that 119 of the genomes contained at least one gene encoding for phytate degradation. However, 27 genomes bore two genes; four genomes harbored four or five genes. The highest number of genes involved in phytate metabolism, six and seven, contain *Lentilactobacillus diolivorans* DSM 14421, *Lp. pentosus* DSM 20314, *Lp. rhamnosus* DSM 20021, *L. aquaticus* DSM 21051, *L. sucicola* DSM 21376, *L. uvarum* DSM 19971, *L. ginsenosidimutans* strain EMML 3041, and *Furfurilactobacillus rossiae* DSM 15814.

### 4.7. Microbial Degradation of Oxalates

Humans lack an endogenous oxalate-degrading pathway and must rely on the gut microbiota or probiotic strains in functional foods. Two key enzymes, oxalyl-CoA decarboxylase (OXC) and formyl-CoA transferase (FCR), mediate oxalate degradation to carbon dioxide and formate.

**Figure 8:**
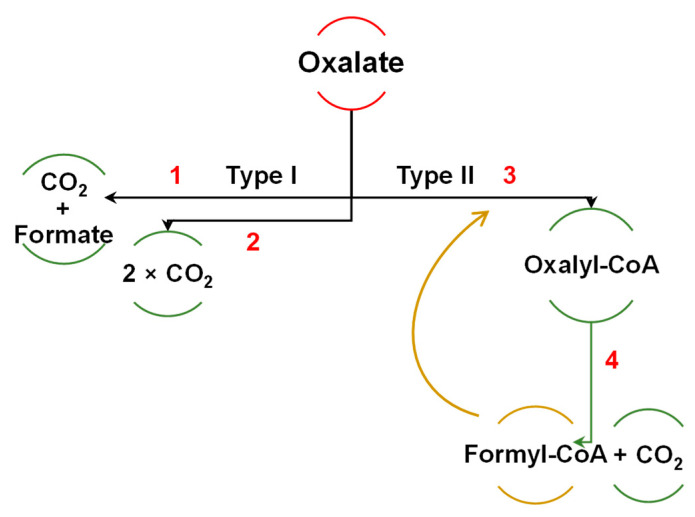
Major pathways of microbial oxalate degradation, according to Kamarad et al. [212], and Azcarate-Peril et al. [213]. Enzymes: 1, oxalate decarboxylase EC 4.1.1.2; 2, oxalate oxidase EC 1.2.3.4; 3, formyl-CoA transferase (FCR), EC 2.8.3.16; 4, oxalyl-CoA decarboxylase (OXC), EC 2.8.3.2.

*Oxalobacter formigenes*, found in the digestive systems of humans and other vertebrates, considerably degrade oxalate, but its application in probiotics designed to prevent kidney failure is problematic. Common probiotic bacteria like *Bifidobacterium* and *Lactobacillus* spp. also degrade oxalates [212,213]. One multi-omics study (>3000 samples from >1000 subjects) shows that human microbiota uses mostly the type II pathway of oxalate degradation, which involves the two-stage process with OXC, FCR, and CoA-intermediates (Figure 8). Less common is the type I pathway in which oxalate is simultaneously converted into two molecules of CO_2_ by oxalate oxidase (EC 1.2.3.4), or one molecule of formate and one molecule of CO_2_ by oxalate decarboxylase (EC 4.1.1.2) [212].

Much about the oxalate-degrading genes and their regulation remains obscure. Bioinformatic analysis of 1396 putative OXC sequences from the UniProt database and 380 human microbiome datasets revealed that OXC may be grouped in seven clusters exhibiting some genetic level variance. OXC and FCR are usually organized in an operon, for example in *Streptomyces* and *Lactobacillus*, but not, curiously, in *Oxalobacter formigenes*. In *E. coli*, *Bifidobacteria*, and *L. acidophilus,* the *oxc* gene is combined with genes encoding cadmium/manganese ATPase transporters [213].

Strong oxalate-degrading ability is not especially common among probiotic bacteria [214]. Of 251 LAB isolates obtained from human feces and south Indian fermented food, only 17 proved able to degrade oxalates significantly (40–63%) [215]. Only nine of them exhibited considerable tolerance to pH 3.0 and 0.3% bile, and of these only three, *Limosilactobacillus fermentum* strains TY5 and AB1, and *L. salivarius* AB11, seem safe enough to be suggested as possible probiotic candidates for alleviating, or even preventing, hyperoxaluria [216].

## 5. Conclusions

A wide range of adverse effects of antinutrients on human health have been extensively documented in recent decades. Conversely, and controversially, some authors have extolled the putative beneficial effects of antinutrients while excluding almost completely their dangerous qualities. Microbial fermentation is a more economical, effective, and versatile approach for ANF reduction in foods, compared to other traditional methods such as soaking or cooking. In the last decade, genetic foundations and biochemical mechanisms of microbial reduction of ANFs in fermented foods attracted much attention. Recent genomics studies showed that the lactic acid bacteria of different genera have the genetic prerequisites to detoxify foods from different antinutrients. Among them, the species *Lp. plantarum* appears as it contains a vast genetic pool for glycoside hydrolase enzymes, including β-glucosidase, tannase, and β-glucuronidase, as well as for a set of enzymes involved in phytate degradation. It turns out that it is advisable to add the ability to degrade antinutrients to the required properties of a certain probiotic.

## Figures and Tables

**Table 1:** Major types of antinutrients.

Antinutrient	Subtypes	Foods	Chemical Nature	Mechanism of Action	Reference
Proteins					
Protease inhibitors	Trypsin inhibitors	Legume seeds	Protein	Inhibit trypsin, chymotrypsin, and protein digestion	[11]
Lectins		Beans, peas, carrots, tomato, potatoes, fruits	Protein	Bind carbohydrates specifically	[2,3]
Phenols					
Tannins	Condensed (catechin, epicatechin, etc.)	Tea, cocoa, grapes, apples, apricots, berries, nuts	Polyflavans	Bind proteins by hydrogen bonds and hydrophobic interactions, thus decreasing iron and calcium absorption; anti-trypsin and anti-amylase activities	[2]
	Hydrolysable (tannic acid, etc.)	Walnuts, pomegranates	Esters of carbohydrates with gallic/ellagic acid	Decrease the bioavailability of non-heme iron; form a complex with vitamin B	[4]
Phytoestrogens	Isoflavones (genistein, daidzein, glycitein, biochanin A)	Soy and soy products	Hydroxylated and methylated isoflavone derivatives; structurally similar to 17-β-estradiol	Binds to the estrogen receptor, modulates estrogenic activity	[22]
	Lignans	Flaxseeds, sesame seeds	Diverse derivatives of phenylpropanoid dimers (C18)	Estrogen receptor and MAPK pathway	[2]
	Stilbenes (e.g., resveratrol; gigantol)	Grapes, peanuts rhubarb	Hydroxylated derivatives of stilbene (1,2-diphenyl ethene)	Modulate NF-β-B, MAPK and JAK/STAT pathways involved in inflammation	[3]
	Coumestans	Lima beans, alfalfa	Hydroxylated and methylated coumestan derivatives; structurally similar to 17-β-estradiol	Binds to estrogen receptor, modulates estrogenic activity	[2]
Gossypol		Cotton seeds	Terpenoid aldehyde	Binds the β-amino group of lysine, limiting its bioavailability	[1]
Glycosides					
Goitrogens		*Brassica* vegetables, millet, cassava	Glucosinolates, derivatives of glucose and amino acids; sulfur-containing	Inhibit iodine uptake	[21]
Cyanogenic glycosides		Cassava, cocoyam (leaves, roots), bamboo (stem, sprouts), sorghum, apples (grains and fruits), apricots (kernels)	α-hydroxynitrile (cyanohydrin) glycosylated with glucose or gentiobiose (glu-β(1→6)-glu)	Hydrolyzed by β-glucosidase to α-hydroxynitrile, which is spontaneously decomposed to HCN and aldehyde/ketone	[10]
Saponins	Triterpenoid	Legumes	Triterpenoid aglycone plus mono- or oligosaccharide glycone	Bind to intestinal cells and minimize absorption and utilization of nutrients	[13]
	Steroids	Oats, pepper, asparagus, ginseng	Steroid aglycone plus mono- or oligosaccharide glycone		[13]
Anti-minerals					
	Oxalates	Spinach, rhubarb, beet greens, amaranth, taro, swiss chard, sweet potatoes	Salts of the oxalic acid (ethanedioic, H_2_C_2_O_4_)	Relatively insoluble salts with Ca^2+^, Mg^2+^, Zn^2+^, and Fe^2+^, limiting their bioavailability	[7]
	Phytates	Legumes, cereals, nuts, seeds, pseudocereals	Salts of the phytic acid (myoinositol hexaphosphate)	Insoluble salts with Zn^2+^, Ca^2+^, Mn^2+^, Mg^2+^, and Fe^2+^, limiting their bioavailability	[14]
Anti-enzymes					
	Solanine	Potatoes, tomatoes, eggplant	Glycoalkaloid, saponin-like	Cholinesterase inhibitor	[1]
	Amylase inhibitors	Wheat, oats, rye	-	Inhibits absorption of dietary starch	[17]
	Arginase inhibitors	Sunflower seeds	-	Inhibits the last step of the urea cycle and the nitrogen cycle	[17]
	Protease inhibitors	Egg white	Ovostatin, ovomucoid, ovoinhibitor, and cystatin	Inhibit digestive enzymes	[17]
Anti-vitamins	Anti-vitamin K Anti-vitamin B7 Anti-vitamin C Anti-vitamin A, E, D Anti-vitamin B1	Alfalfa, grapefruit Eggs Melon, squash, zucchini, cucumber Soybeans Raw fish	Coumarins Avidin Ascorbic acid oxidase Lipoxidase Thiaminase	Inhibitors or structural modifiers of vitamins that interact with enzymes, interfering with their natural functions; or are vitamin-destroying enzymes	[3]

**Table 2:** Proanthocyanidins food sources, according to Smeriglio et al. [98].

Food/Plant	Latin Name	Concentration (mg/100 g, or mg/100 mL)
Cacao beans	*Theobroma cacao* L.	6100–8100
Grape seeds	*Vitis vinifera* L.	2180–6050
Sorghum	*Sorghum bicolor*	413–5333
Chokeberries	*Aronia melanocarpa*	553–2106
Chocolate		828–1332
Beans	*Phaseolus vulgaris* L.	5–830
Pecans	*Carya illinoinensis*	238–695
Hazelnuts	*Corylus* spp.	125–645
Lingonberries	*Vaccinium vitis-idaea* L.	175–545
Cranberries	*Vaccinium oxycoccus* L.	194–496
Blueberries	*Vaccinium corymbosum* L.	311–335
-	*Vaccinium myrtillus* L.	87–274
Apple	*Malus domestica*	46–278
Almonds	*Prunus dulcis*	67–257

**Table 3:** Experimental evidence on adverse effects of antinutrients.

ANF	Source	Suggested Effects	Experimental Effects	Model	Reference
Lectins	Beans, peas, carrots, tomato, potatoes, fruits, lentils, wheat, soybean, peanuts, gorse, *Artocarpus integrifolia*	Altered gut function, inflammation; systemic metabolism disruption; key organ damage	ND	ND	[50]
			Activation of caspase-1 and IL-1β via the NLRP3 inflammasome	Mice	[44]
Tannins	Tea, cocoa, grape, berries	↓ Iron absorption and stores	ND *	ND	[2]
	Tea		54% with low Hb levels (with tea); 69% with normal Hb levels (*w*/*o* tea)	Humans	[107]
	Sorghum, Tannic acid		Dose-dependent decrease in milk production with increased tannic acid in the diet	Cows	[108]
	Tea		Correlation between tannin intake and low levels of Hb and transferrin receptor	Humans (stunted overweight teenagers)	[109]
Goitrogens	*Brassica* vegetables, millet, cassava	↓ Iodine uptake; hypothyroidism and goiter	ND	ND	[2]
	Cruciferous vegetables diet		1.5-fold higher risk of thyroid carcinoma	Humans	[73]
	Cruciferous vegetables, rich in brassicas diet		High prevalence of thyroid cancer, mild iodine deficiency	Humans	[74]
Phytoestrogens	Soy and soy products	Endocrine disruption, higher risk of estrogen-sensitive cancers	ND	ND	[2]
Cyanogenic glycosides	Cassava, cocoyam, bamboo, sorghum	Generate HCN, a potent respiratory inhibitor	ND	ND	[56]
	Cassava	ND	Growth retardation in children	Humans	[66]
Saponins	Legumes (triterpenoid) Oats, pepper, asparagus, ginseng (steroid)		ND	ND	[55,85]
	Bitter leaf (*Vernonia amygdalina*)	ND	Hemolytic effect on human erythrocytes	Humans	[88]
	Commercial saponin	ND	Hemolysis and apoptosis of erythrocytes due to increased Ca^2+^ and ceramide formation	Humans	[89]
Oxalates	Spinach, rhubarb, beet greens	↓ Ca^2+^ absorption; promotion of kidney stones and failure	AGXT linked with decreased glycine/oxalate ratio and risk of atherosclerosis -	Mice Humans Macrophage	[123,124]
Phytates	Legumes, cereals, nuts, seeds	↓ Ca^2+^, ↓ Fe^2+/3+^, ↓ Zn^2+^ absorption	ND	ND	[2]
	Phytic acid added to white bread		↓ Mg absorption (dose-dependent)	Humans	[114]
	Common beans (*Phaseolus vulgaris*), also rich in polyphenols		14–45% ↓ Fe absorption, not improved unless both phytates and polyphenols were removed	Humans	[115]

* ND, no data available.

**Table 4:** Microbial degradation of antinutrients.

ANF *	Source	Species/Strain	Initial Concentration	Maximum Reduction (%)	Fermentation Conditions	Reference
Lectins	Lentils	Unspecified starter culture	79 g/L	98%	72–96 h, 42 °C	[163]
Cyanogenic glycosides	Linseed	*L. acidophilus* MTCC-10307	5.17 mg/g	66%	2 days, 30 °C	[164]
Goitrogens	Raw white cabbage	*Leuconostoc mesenteroides*, *E. faecium*	168 μmol/100 g FW *	~90% 100%	5 days, 20 °C 7 days, 20 °C	[165]
Saponins	Quinoa dough	*Lp. plantarum* 1	272 mg/100 g	64% 71%	3 days, 35 °C 4 days, 35 °C	[166]
Tannins	Goat feces	*Klebsiella pneumoniae*	64 g/L	68% 98%	24 h, 30 °C 72 h, 30 °C	[167]
Trypsin inhibitors	Soybean	*Lev. brevis* CICC 6004	6.4 mg/g DW *	57.1%	72 h, pH 5.1, 10% inoculum	[168]
Amylase inhibitors	Pearl millet	Unspecified starter culture	n/a	50.8%	24 h, 30 °C	[169]
Phytates	Tarhana	*Fructilactobacillus sanfranciscensis* CCM 7699	3.3 g/kg	89%	144 h, pH 4.8–4.2	[170]
Oxalates	Ammonium oxalate	*L. acidophilus*	10 mM	11.8%	48 h	[171]
	Ammonium oxalate	*Streptococcus thermophilus*	10 mM	3.5%	48 h	[171]
	Sodium oxalate	*Enterococcus faecalis*	5 g/L	100%	48 h	[172]

* Abbreviations: ANF, Antinutritional Factor; DW, dry weight; FW, fresh weight.

## Data Availability

No new data were created or analyzed in this study. Data sharing is not applicable to this article.
